# A Novel Option of Uninterrupted Closure of Surgical Wounds

**DOI:** 10.4103/0974-2077.58520

**Published:** 2009

**Authors:** Marlen A Sulamanidze, George M Sulamanidze

**Affiliations:** *Clinic of Plastic and Aesthetic Surgery, Total Charm, Moscow, Russia*

**Keywords:** Scars, sutures, APTOS

## Abstract

**Background:**

A cosmetically pleasing postoperative scar is an important aim of all aesthetic surgeries. Use of proper suture materials for delicate and gentle suturing of the operative injury is an important requirement for achieving satisfactory scars. However, closure of the edges of wounds by means of conventional suture materials does not always meet the requirements to achieve this objective.

**Aim:**

To simplify and facilitate the process of surgical wound closure, to improve the quality of scar, and to achieve a good cosmetic effect through the introduction of a new type of suture material.

**Materials and Methods:**

We have introduced a new surgical suturing material—a nontraumatic, barbed thread connected with the suture needle—APTOS SUTURE (European patent 1075843 as of 1999). Presented herein is a new modification of the technique of uninterrupted subcutaneous and intracutaneous suturing of wound edges, and the details of our experience with this material.

**Results:**

Our experience shows that, with use of APTOS, wound closure is carried out easily and quickly. The wound remains stable, the time of healing is shortened, and the process of suture removal is simplified, resulting in an aesthetically pleasing scar.

**Conclusions:**

The technique of surgical wound suturing proposed herein is a simple, facilitated, and efficient option of wound-edge closure, which can successfully be used, both in general and in aesthetic surgery for wound closure, such as plasty of scars, face lift, mammoplasty, and abdominal plasty.

## INTRODUCTION

An aesthetically pleasing scar should be thin, lying at the level of the skin, differing little in color or density, and not leading to any contractures or displacement of the surrounding tissues. Ideally, closure of wounds' edges should result in a scar that is shorter than the initial incision.[1‐9]

Important factors for obtaining a good postoperative scar include: High-quality suture material to close the wound's edges, appropriate methods of subcutaneous and intradermal uninterrupted suturing, and appropriate approximation of the wound's edges until the scar becomes sufficiently strong, when the suture itself no longer remains necessary.[2‐6,8‐10]

Previous studies have proved that there is a complex interrelationship, both biological and mechanical, between the thread and the tissue.[11,12] Besides being needed to approximate the edges of the wound to facilitate healing, it also introduces a foreign medium, contributing to inflammation. The presence of suture material in the wound promotes an increase in the wound's strength but only for 9-15 days. Thereafter, it loses its significance as it resists healing and hence, the thread should be removed as soon as possible.[3,5,6,9,10,13‐15] Removal of the irritating element is associated with the elimination of microorganisms brought from the skin's surface, and therefore, a decrease in the incidence rate of suppuration. It also accelerates healing due to lower tissue compression, better blood and lymph flow, and improved immunobiological and bactericidal properties of tissue. Early thread removal is associated with shortened duration of treatment due to decreased inflammation in the wound and quicker keratinization of the epidermis which serves as a good barrier to pathogen invasion. Hence, a primary requirement to produce a cosmetically satisfying postoperative scar is early suture removal.

Aesthetic surgeons have long been using atraumatic suture material for the uninterrupted closure technique. There have been several improvements in suture needles and threads, leading to the introduction of threads with a smoother surface. It is believed that a smooth thread easily slips within tissue, thereby causing less injury to the surrounding tissues, is more convenient to use, and can be easily pulled out and removed from tissue.

However, despite these improvements, closing the wound's edges with conventional smooth suture material does not always comply with the present-day requirements of an ideal postoperative scar because of the following limitations:

- At the beginning and the end of the wound, the thread's ends have to be fixed by means of one of the established techniques, usually with noose sutures.
- These noose sutures, at the beginning and the end of the wound, leave transverse coarse scars after removal.
- While suturing, the surgeon needs the help of an assistant, to keep the thread tightened while applying each stitch.
- While closing a long wound, the tissue slides along the thread's length from the middle, being bunched to some degree at the edges, which eventually results in deterioration of the scar's quality.
- While removing a suture, one has to release the thread, at the beginning and the end of the wound, from the thread's nodes, with the ends of a long thread sometimes slipping under the skin. This complicates thread removal, or sometimes makes it impossible.
- Conventional sutures are also time-consuming, both to apply and to remove.

Barbed suturing threads have been introduced to overcome these disadvantages and limitations. An earlier attempt sought to use barbed surgical suture materials to close the wound's edges with perpendicular single stitches (US patents *N*o. 5053047 as of 01.10.1991, *N*o. 5342376 dated 30.08.1994, and WO-A-98/52473 as of 21.05.1997). This, however, was not successful due to several limitations such as unaesthetic postoperative scars, unreliable subcutaneous and intradermal closure of the wounds' edges, and cumbersome nature and high cost of both the suture material and the appliances required.

In 2006, a group of workers from the USA[16] suggested the use of uninterrupted subcutaneous and subdermal wound closure by resorbable barbed suture material created according to patent PCT/US 2003/030424. In Russia, in 1996, we proposed surgical suture material APTOS suture made from nonabsorbable material for wound closure with an uninterrupted subcutaneous or intradermal suture. This suture material has received approval and certification from both Russian and European authorities.[8,9,14,15]

## MATERIALS AND METHODS

### Design and mechanism of the non-absorbable APTOS suture

**Figure 1:**
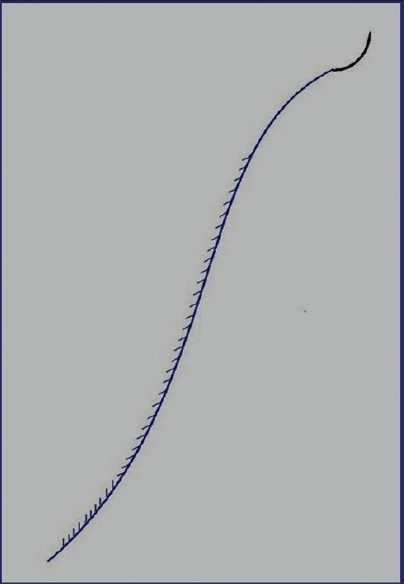
Schematic view of APTOS suture

The surgical needle for this suture material is as usual – curved, atraumatic, with the suture thread connected to it. The thread is made of monofilament polypropylene (manufactured by the G. Krahmer GmbH, Germany) with barbs. These barbs are sequentially positioned along the length of the thread and are inclined, with pointed edges directed opposite to the thread's pulling through. This is the first set of barbs, which after the wound closure, remains within the tissue and is responsible for qualitative approximation of the wound's edges. At a distance of about 5-7 cm from the free end of the thread, the direction of the barbs is changed with their pointed tips being now oriented in the opposite direction. This is the second set of barbs, which after wound closure, remains above the skin and is necessary for counteracting the slipping of the thread's end under the skin [Figure 1].

The barbs of the first set are on only one side of the thread, with the interval between them being within five diameters of the thread. The second set of barbs are also on one side along the thread's section, being however positioned much more frequently, *i.e*., the interval between them not exceeding 1.5 diameters of the thread.

**Figure 2:**
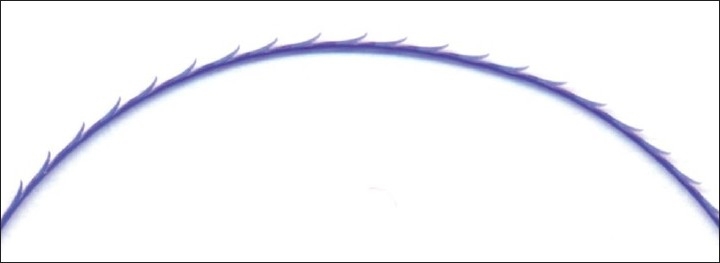
Design of barbs, zoomed in on thread

The barbs of both the first and second sets are made as dents on the thread. The design of the barbs are specifically designed as follows [Figure 2]: The tip of the barb is pointed, deviated beyond the thread's edge; then the barb is slightly thickened, but further on, its thickness decreases gradually. The total height of the barb lies within 1.5-3.0 diameters of the thread. Such a design of the barb is necessary for its better engagement with tissue to counteract any opposing force to withdraw the thread in the opposite direction. The mechanism of action of the barbs of such shape is as follows:

While pulling the thread in the opposite direction with a gradual increase in pressure upon the barb, its tip is slightly elevated, the tissue pressure moves to its thickened portion and the barb becomes elevated even more. Then the pressure gradually moves to a weaker portion of the barb near its base and simultaneously becomes weaker on the thick portion of the barb. It is this factor that leads to the bending of the barb in a manner similar to that of the fish-hook and is responsible for reliable engagement with tissue.[15]

The overall length of the barbed thread is 25 cm, with the ratio of the first- and second-set portions of the thread being approximately four to one, *i.e*., the first working part approximately amounting to 15 cm. The use of a longer APTOS suture for wound closure in one span is neither appropriate nor advisable as its removal from the wound is fraught with difficulty. This principle on the desired length of suture is applicable to traditional sutures (with a smooth thread) also and is therefore not specific for APTOS sutures.

An improvement of this suture is the introduction of double-length thread consisting of two similarly equal parts, like a mirror reflection, with needles at both ends. After opening the sterile pack, such suture material may be cut into two equal parts to be used to close two different wounds in the same patient, or for closing the same wound but in two levels, or two different portions of the wound from its middle towards the ends. In the last case, there is no need to cut the thread into two parts.

### Wound closure with APTOS suture

To close a wound with an uninterrupted cosmetic suture, the operator introduces the needle about 1 cm from any of the wound's ends, with the needle's pointed end then emerging in the subcutaneous fat of the wound. The needle with the thread is pulled into the wound as far as it will go, *i.e*., until the second set of front barbs oriented in the opposite direction would abut against the skin's surface in the place of the thread entrance. These barbs prevent further movement of the thread under the skin, with the thread's end being 5-7 cm long above the skin's surface. Further on, uninterrupted stitches are applied by the usual method, alternately upon each of the wound's edges, gradually advancing towards the opposite end of the wound, with the thread easily slipping towards the side of suturing but not moving in the opposite direction. With each stitch applied, the thread should be tightened with some extra strength. This would result in approximating of the wound's ends and a slightly pronounced crimping of tissues, thus preventing the sutured portion of the wound from diverging. When completing the wound closure at the opposite end of the wound, it is necessary to bring the needle to the surface of the skin, approximately 1 cm from its end. The thread is then brought to the skin's surface, additionally pulling it on and cut it off to leave a thread's portion of not less than 5 cm length above the skin. The barbs of this thread's portion (the second end) will prevent the thread from slipping under the skin in the opposite direction. Thus, the oppositely oriented barbs of the first and second thread's ends, would remain above the skin at the opposite ends of the wound. Thus, the wound's edges will be held tightly in the sutured position, thus completing uninterrupted closure of subcutaneous fat.

**Figure 3:**
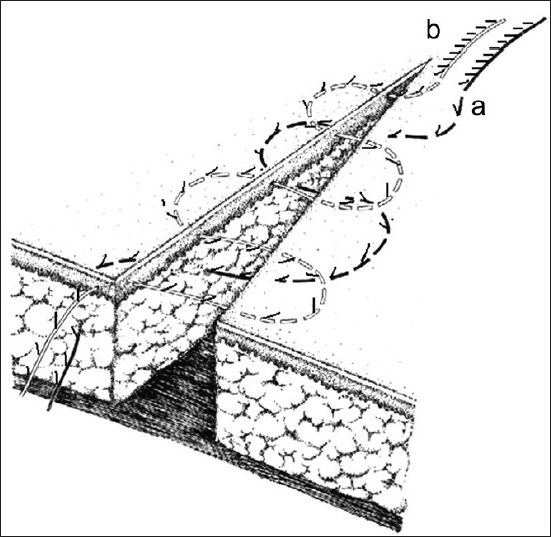
Version of wound edge suturing in two levels using subcutaneous and intradermal methods in application with single suture thread (layout)

Uninterrupted intradermal suturing is performed in a similar manner by the same technique. Figure 3 shows an option of closing a wound's edges in two rows by means of subcutaneous and intradermal methods by using monofilament suture threads.

**Figure 4:**
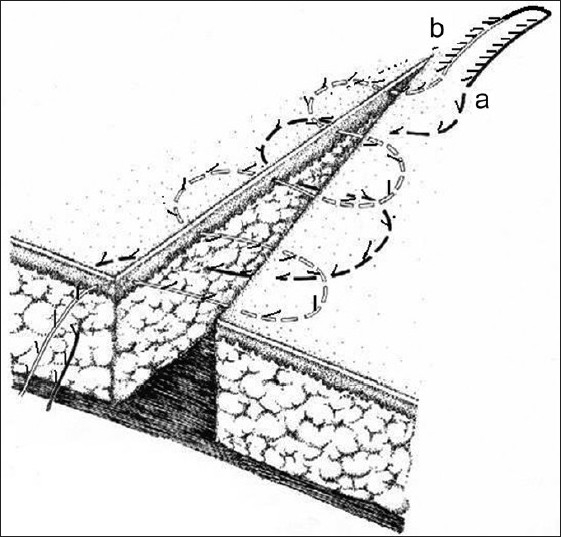
Version of wound edge suturing in two levels using subcutaneous and intradermal methods in application with double suture thread (layout)

Figure 4 shows a variant of closing a wound's edges in two levels by subcutaneous and intradermal methods by using double-filament suture threads.

A variant of closing the edges of a long wound using a two-filament thread from its middle towards the opposite sides is also possible.

### Removal of APTOS sutures

After appropriate antiseptic treatment of the wound, the thread's end is pincer-pulled from the beginning of the wound suture and is cut off in the place where the barbs change their orientation [Figure 1]. Then, from the opposite side, the whole thread is pulled out from the subcutaneous space, which is easy to perform due to folding of the barbs.

The thread's end that needs to be cut off differs from the opposite end of the thread which is used to pull out the whole thread by more frequently located barbs. Therefore, the operator who has to retrieve the sutures applied by his/her colleague would easily determine the manner to remove the sutures.

### Study design and study cohort

A preliminary study was carried out in our clinic with this product from May to October 1998. We studied the efficacy of barbed suture threads made of (3 metric) 2/0 polypropylene monofilament for cosmetic wound closure. The needles were 25 and 30 mm long and the curvature was 3/8 as compared with conventional smooth threads having otherwise similar properties. A total of 38 patients undergoing a variety of plastic operations on the trunk, face, and limbs (abdominoplasty, mammoplasty, cicatricial plasty), were recruited for this study. The wounds were closed using either APTOS suture threads or conventional sutures. Of these 38 patients, 25 patients had their wounds sutured with the APTOS suture and 13 with traditional suture material. Wound closure was performed by the conventional technique for the traditional nonbarbed sutures and by the method described above for the APTOS suture.

Assessment was subjective and done by the surgeon during the procedure, based on the ease of administration. Objective assessment involved the speed of suturing and the stability of the sutured wound, both during the operation and postoperatively, tissue reaction to the thread (edema, infiltration, regeneration), ease of removal of the thread once the wound had healed, and most importantly, the resultant scar's condition.

## RESULTS

Subjective assessment while dealing with APTOS suture threads for wound closure showed that the manipulations were easy to perform, requiring no anchoring of the thread's ends at the beginning and the end of the wound, thereby shortening the duration of closure and simplifying the procedure. The surgeons reported simplicity while removing such sutures as well. Neither the wound's stability during the operation and postoperatively, nor the reaction of tissues to the thread changed. Wound healing occurred uneventfully with no traces of the noose sutures at the beginning and end of the wound, resulting in the better appearance of the scar. Besides, we observed a 10-15% shortening of the wound in the immediate postoperative period, although no deeper study of this phenomenon was conducted in later periods.

There was also another important advantage: Usually, in conventional suture methods, drainages are introduced through additional cuts or incisions and not through the wound's edges. This method results in an additional, coarse cicatrix. With APTOS sutures, the drainage can be inserted in the wound itself so that the end of the thread is pulled out during removal. Thus, the edges of the wound from the drainage are easily put together and the quality of the scar in this place does not differ from the primary one. Thus, the outcomes in the immediate and later postoperative periods were satisfactory.[8]

## DISCUSSION

**Figure 5:**
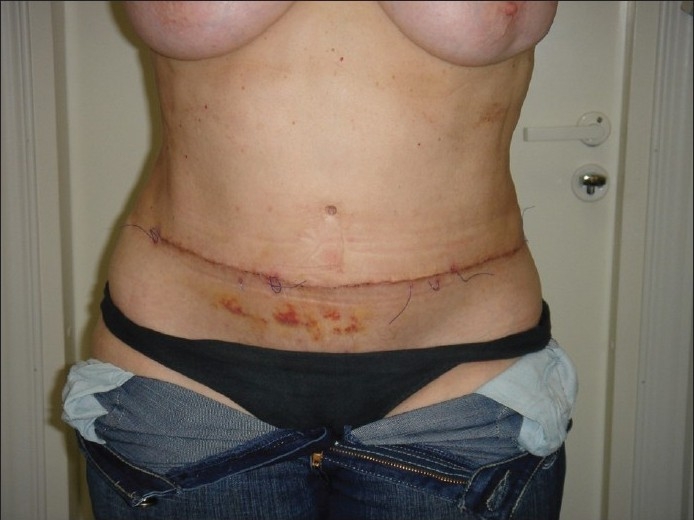
Case of use at coarse scar after abdominoplasty

Over the past seven years (from January 2000 to January 2007), we have used APTOS suture materials and the wound closure technique described above for a total of 435 cases. Figures 5‐8 show several case reports; Figure 9 shows a list of operations in which we used this method of wound closure and their percentage of the total number.

**Figure 6:**
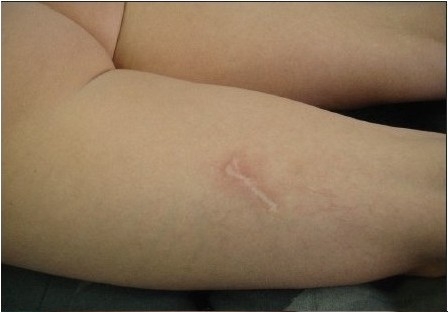
Case of use at scar debridement; before

**Figure 6a:**
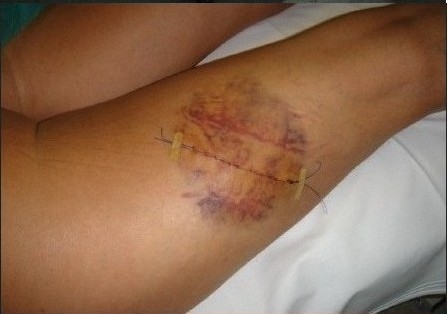
Case in Figure 6, 2 days of scar revision with APTOS suture

**Figure 6b:**
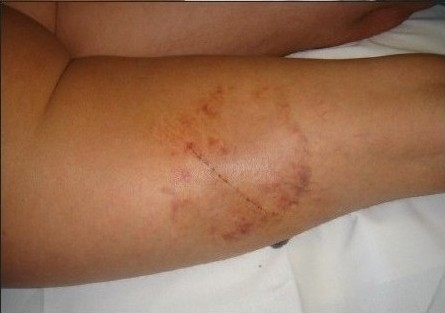
Case as in Figure 6 at the moment of suture removal

**Figure 7:**
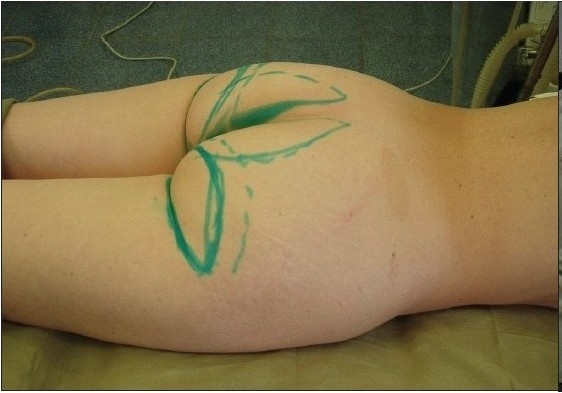
Case of use at gluteoplasty; before

**Figure 7a:**
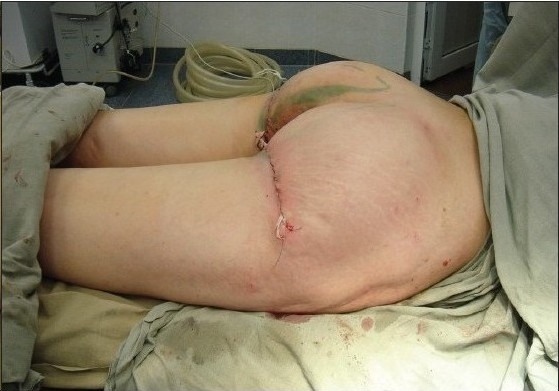
Case of gluteoplasty after the operation

**Figure 8:**
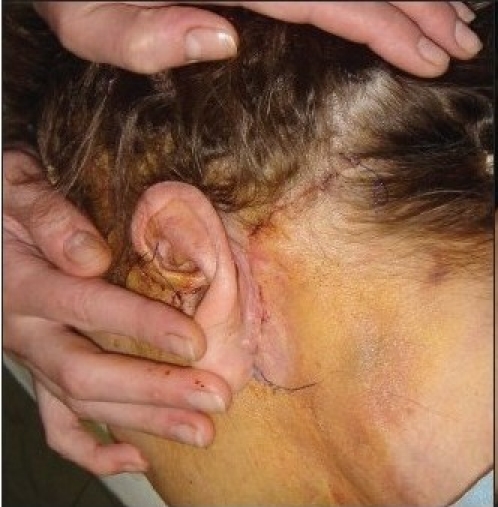
Facelift at the end of surgery

**Figure 8a:**
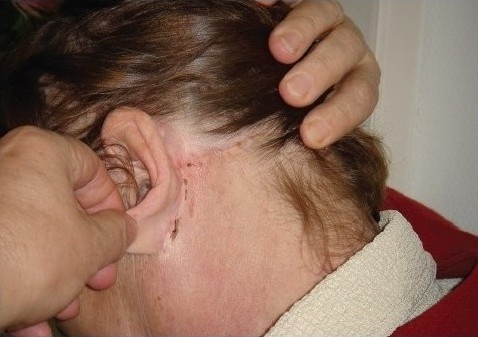
Facelift before suture removal

**Figure 8b:**
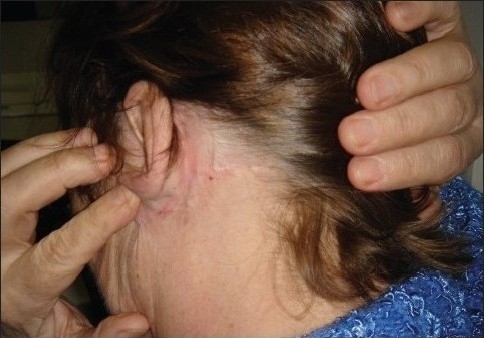
Facelift 20 days later

**Figure 9:**
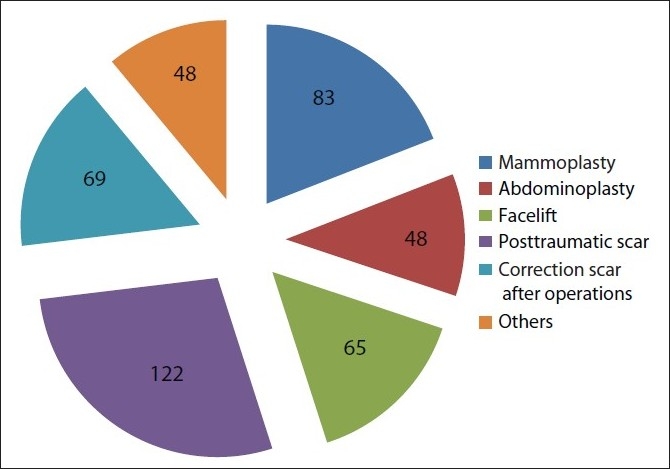
List of operations where this method of wound suturing was used and its percentage of the total number

### Limitations of APTOS suture

However, during subsequent use, certain limitations were noted:

1. Sometimes at the beginning of the closing procedure, the barbs on the counteracting end of the thread failed to withstand the pressure of the pull and would slip under the skin, weakening the wound's edges in that spot. To prevent this end from completely slipping under the skin, we recommended to the manufacturer that this portion of the thread be elongated from 5 to 7 cm and that the barbs be reinforced. In addition, surgeons were also advised to be gentler while tightening the thread when making the initial suture.
2. If the wound was sutured in two levels and the sutures are to be removed by another operator, the latter would find it difficult to decide which ends belong to the thread of the upper and which to the lower level. To eliminate this difficulty, we stuck to the following principle: The ends of the thread used to close the deeper row (*e.g*., subcutaneous) are pulled out caudally to the length of the wound, while the ends of the superficial sutures (*e.g*., intradermal) are to be pulled out cranially. Guided by this principle, a surgeon having to remove sutures placed by his/her colleague, could easily determine which of the sutures has to be retrieved first.
3. Pointed barbs of the thread's ends, both at the beginning and at the end of the wound, tend to catch on the gauze bandage applied onto the wound, which makes postoperative dressing inconvenient. Therefore, immediately on completion of the operation, the threads' ends are glued to the skin with pieces of sterile adhesive tape.

In the current stage of trials, APTOS threads were made only of nonabsorbable materials because tissue response to absorbable threads is known to be more pronounced and can result in complications such as hematomas, seromas, infiltration of the wound's edges, granulomas, infection, and ligature fistulas. Such complications are unacceptable as they led to the slowing down of the process of scarring, which in the long run, results in a poor-quality cicatrix. Even under the most favourable conditions, cases of using absorbable material for intradermal wound closure are accompanied and followed by inconveniences: The process of advancing the thread through the skin thickness while suturing is difficult, as all absorbable materials possess a high coefficient of friction and slide badly within tissues. Therefore, absorbable threads should not be used for intradermal sutures that need to be removed. Removal of an intradermal suture is of principal importance also because if left within tissue, it may result in visually noticeable, dark portions of the scar.[3,5,6,9,10,13,15] Absorbable suture material is used only in deep layers to remain there until resolved completely.

## CONCLUSIONS

1. The process of wound closure with an uninterrupted suture by using the barbed thread according to our technique takes less time, for there is no need for anchoring the ends of the threads (noose sutures) at the beginning and at the end of the wound.
2. While suturing with conventional threads, the stitches of a smooth thread slip in the punctured canal and do not hold the wound's edges put together. Hence, the thread needs to be pulled constantly and maintained in a strained position, which requires assistance. Unlike smooth threads, the barbed suture material makes it possible to tighten and to bring together the wound's edges with each stitch, not allowing them to diverge and thus, removing the need for assistance.
3. Some extra, yet gentle, pulling of the thread while applying each stitch at the end of the procedure results in uniform approximation of wound margins, leading to even distribution of tension along the length of the wound and probably to a shorter scar.
4. As no noose sutures are applied at the beginning and at the end of the wound, the postoperative skin bears no traces of otherwise transverse scars.
5. The presence of barbs on the thread makes it possible to perform additional pulling of the thread, thus allowing for due adjustment of the degree of convergence of the wound's edges during redressings. This valuable property of wound closure with barbed threads also permits wound drainage without additional incisions.
6. The process of suture removal is simple and less painful as compared to conventional closure techniques as it is free from the need to retrieve noose sutures at the beginning and at the end of the wound.

Hence, a conclusion could be drawn that the APTOS suture thread makes it possible to efficiently close wounds and thus, may safely be recommended to be used in plastic reconstructive and aesthetic surgery for wound closure with uninterrupted subcutaneous or intradermal sutures.
